# Development of a TB vaccine trial site in Africa and lessons from the Ebola experience

**DOI:** 10.1186/s12889-020-09051-3

**Published:** 2020-06-26

**Authors:** G. Kaguthi, V. Nduba, P. Rabuogi, D. Okelloh, S. G. Ouma, G. Blatner, S. Gelderbloem, Ellen M. H. Mitchell, Cherise P. Scott, S. Verver, T. Hawkridge, J. E. M. de Steenwinkel, K. F. Laserson, J. H. Richardus

**Affiliations:** 1 Centre for Respiratory Diseases Research-Kenya Medical Research Institute (KEMRI-CRDR), Nairobi, Kenya; 2 Department of Public Health, Erasmus MC, University Medical Center Rotterdam, Rotterdam, The Netherlands; 3 (at the time of the studies) KEMRI and Centers for Disease Control and Prevention Public Health Collaboration, Kisumu, Kenya; 4 AERAS (at the time of the studies), Cape Town, South Africa; 5 AERAS (at the time of the studies), Rockville, Maryland USA; 6 Institute of Tropical Medicine, Antwerp, Belgium; 7 (at the time of the studies) KNCV Tuberculosis Foundation, The Hague, The Netherlands

**Keywords:** Vaccines, Ebola, Tuberculosis, Trials, Sites, Emerging infectious diseases

## Abstract

Tuberculosis is the deadliest infection of our time. In contrast, about 11,000 people died of Ebola between 2014 and 2016. Despite this manifest difference in mortality, there is now a vaccine licensed in the United States and by the European Medicines Agency, with up to 100% efficacy against Ebola. The developments that led to the trialing of the Ebola vaccine were historic and unprecedented. The single licensed TB vaccine (BCG) has limited efficacy. There is a dire need for a more efficacious TB vaccine. To deploy such vaccines, trials are needed in sites that combine high disease incidence and research infrastructure. We describe our twelve-year experience building a TB vaccine trial site in contrast to the process in the recent Ebola outbreak. There are additional differences. Relative to the Ebola pipeline, TB vaccines have fewer trials and a paucity of government and industry led trials. While pathogens have varying levels of difficulty in the development of new vaccine candidates, there yet appears to be greater interest in funding and coordinating Ebola interventions. TB is a global threat that requires similar concerted effort for elimination.

## Background

Tuberculosis is a blight to the technological advances of the twenty-first century. A global blueprint to combat it has been outlined in the end TB Strategy [1]. New drugs, diagnostics, and vaccines are in development with the goal of elimination. To demonstrate efficacy and for external validity, trial sites in endemic areas are needed to advance the various candidates through phase III licensure trials. Although there are more than 22 high burden countries (Global TB Report, 2017), vaccine trials occur in only a handful of these, due to inadequate clinical trials capacity. These challenges hinder advancement of TB vaccine candidates.

From 2014 to 2016, there was an outbreak of the Ebola virus in West Africa. Outbreaks had been documented periodically from 1976, with case fatalities of 90%. However, in the recent outbreak, the risk of widespread contagion from global travel and its potential for bioterrorism, prompted strong responses. In a highly entropic environment, 13 prophylactic vaccines were in clinical studies with at least 40% evaluated on the African continent between 2014 and 2015 [2]. This demonstrated that under sufficient threat, the global community can mobilize resources and overcome adverse circumstances. Determining vaccine efficacy during an unpredictable outbreak, in low resourced settings for an acutely lethal disease is singularly difficult. ‘However, there now exists a licensed vaccine which demonstrated up to 100% efficacy’ [2].

In light of these unusual events, we compare the TB and Ebola vaccine development in two ways. Firstly, we chronologically narrate our experience and learning in building a TB vaccine trial site from the ground up. Thereafter, we compare this with the site set-up process for Ebola vaccine trials. Finally, we review the number of clinical vaccine trials for both diseases, by phase and funding, from 2014 when the Ebola outbreak began and Phase I trials were initiated. This review incorporates our unique site experiences in relation to current and future TB vaccine trial sites and places our perspectives on TB vaccine development in the context of the Ebola outbreak.

### TB vaccine trial site development

#### Study area and study population

**Fig. 1:**
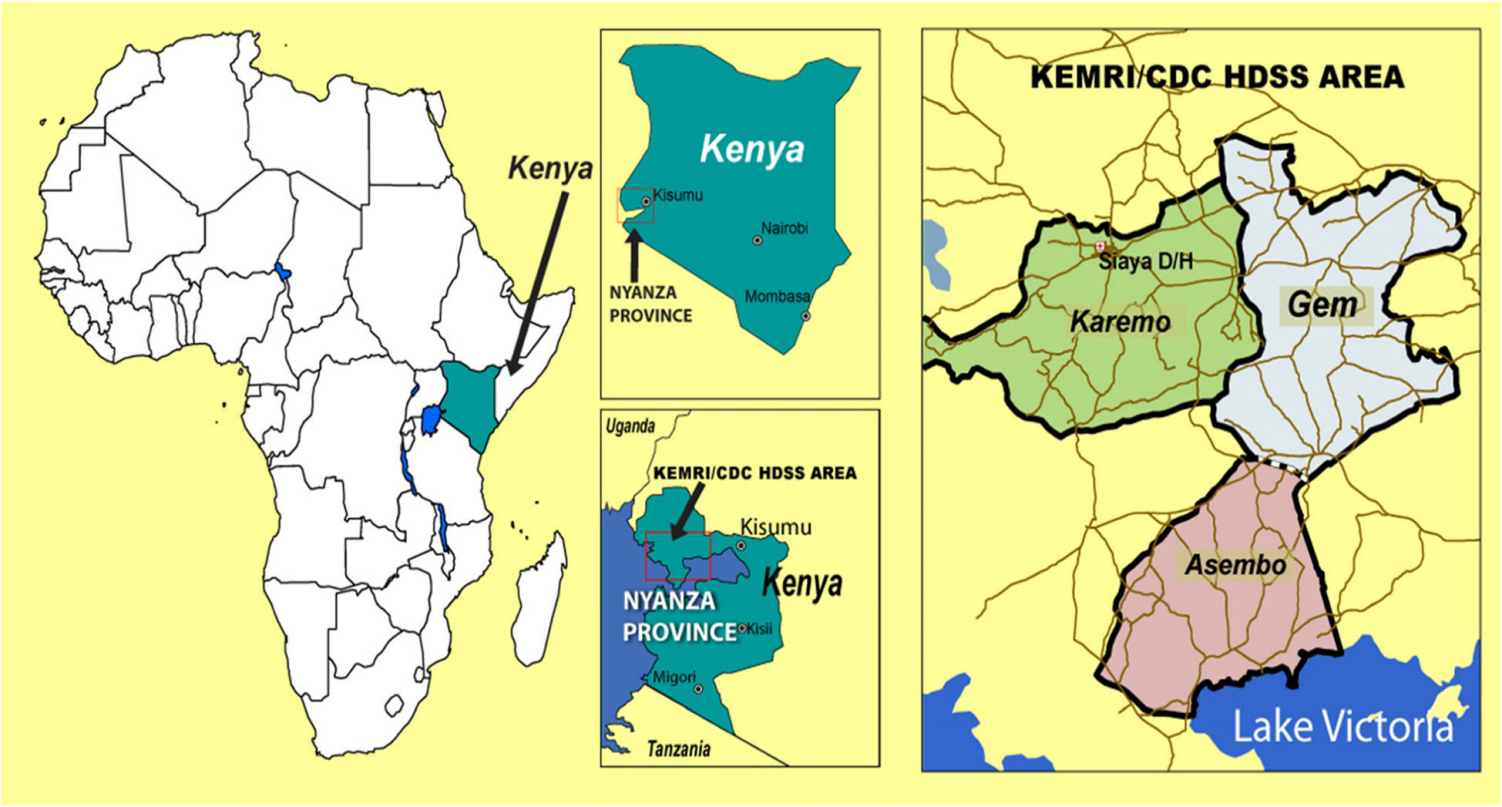
Study area. KEMRI/CDC: Kenya Medical Research Institute/Centers for Disease Control. HDSS: Health and Demographic Surveillance System

Our reflections are based on studies conducted from 2007 to 2019 at a site in Siaya county, Western Kenya (Fig. 1). The area is rural, 400 km west of the capital Nairobi. The population mostly comprises peasant farmers. There is a high but declining prevalence of TB, HIV and malaria [3]. Part of the study area was under health and demographic surveillance system (HDSS), tracking births, deaths, and migrations [3].

### Site history and development

In 2007, we, alongside other collaborators in Mozambique, South Africa and Uganda received an European and Developing Countries Clinical Trials’ Partnership (EDCTP) grant to create scientific and infrastructure capacity necessary for conduct of TB vaccine trials, also for site to site skills transfer.

Two epidemiological TB studies in adolescents and infants were conceived. At the time, there was little research infrastructure. A collaboration with the then Siaya Hospital was set up. Additional training for nurses on TST administration and reading, collection of induced sputa and gastric aspirates was provided by a team from a sister site at the South Africa Tuberculosis Vaccine Initiative. Study staff were extensively trained in collaboration with the Vienna School of Clinical Research on (among others) biostatistics, research methods, ethics, Good Clinical Practice (GCP), and trial site management. Also, several site staff received concurrent sponsorship for MSc’s and PhD’s.

### Tuberculosis studies

We describe five TB studies conducted serially at our site and the lessons learnt in each.

#### Adolescent cohort study (ACS)

The ACS began in 2007, with the objective of recruiting adolescents to establish the optimal way to access them in the study area and determine TB prevalence and incidence in preparation for TB vaccine trials. A mobile field site (MFS) was used to enroll adolescents. (Supplementary Figure 5-Mobile Field Site). The MFS was a collection of tents, a mobile radiography unit and mobile generator pitched in a grassy open field in a school, church compound or other open public arena. Potential MFS sites were identified by study staff, and the site assembled every week. The tents housed study staff, parents and adolescents who moved serially from consenting, TB symptom screening, TST administration or reading, to chest radiography and then to dispatch. Data entry was on Personal Digital Assistants and laptops, using a local area network powered by the mobile generator. Upon exhausting all potentials at that catchment area, the site would demobilize and set up in a contiguous site and the process would start over again. Follow-up visits were conducted in similar fashion, from site to site. The challenges of operating in an open field were particularly apparent during the rainy seasons. The site would get flooded or barely accessible, disrupting study processes. In that case it would be disassembled and relocated. A total of 5004 adolescents were recruited on target over 12 months and 83% retained [4]. Most of the study team rolled over into the infant study.

#### Infant cohort study (ICS)

Pediatric TB estimates from program data were not age-disaggregated, masking the disproportionate affliction of young infants with TB. In 2008, the thinking of the TB vaccine community leaned heavily towards infants as a key efficacy target population [5]. Therefore, the ICS was conceived to provide estimates of TB incidence using comprehensive diagnostic methods [5], and to demonstrate capacity to deliver BCG within 96 h of birth. This was to anticipate the administration of BCG replacement candidate vaccines.

At the time, it was reported that 80% of mothers delivered at home, with the help of a traditional birth attendant (TBA). We engaged TBAs to assist in notifying births to the study staff. The recruitment coordinator would then dispatch a nurse on a motorbike to perform enrolment procedures. Thereafter, a nurse would deliver BCG to enrolled infants. The median age of enrollees was 10 days, therefore in many cases it was not possible to deliver BCG within 96 h of birth due to late notifications and problems locating mothers who were highly mobile in the study area. This was accentuated when due to insufficient notifications, the study area was extended to Boro, a nearby sub-county, which was not under the HDSS.

In addition, the terrain was frequently challenging. Nurses were inexperienced motorbike riders and predominantly female. There were several falls that injured their morale more than anything. Nevertheless, the team rallied, and 2900 infants were enrolled on target within 12 months.

Fortunately, the number of home births in the study area continued to decline. By 2011, at least 40% occurred at health facilities [6]. Future trials of BCG replacement vaccine trials will therefore enroll infants at health facilities.

The ICS operated out of two tents in a grassy field of the Siaya Hospital, seeing scheduled and unscheduled visitors. The hospital allocated a store in the pediatric ward for creation of a negative pressure room for specialized sputum collection procedures. We renovated a defunct, dilapidated amenity wing designed for private patients, and converted it into a case verification ward for overnight admissions for TB investigation procedures.

The existing hodgepodge of facilities did not meet trial requirements, as there was no GCP compliant pharmacy, archival facilities, or laboratories. Therefore, in 2009, construction of a state-of-the-art research annex (Supplementary Figure 4-Siaya Clinical Research Annex) with all the requisite infrastructure at the hospital grounds began, funded by several partners. It was completed in 2012 and auspiciously launched.

The study also created a previously non-existent capacity to diagnose pediatric TB using induced sputa and gastric aspirates as well as structured chest radiograph review. Staff training for the latter two procedures were provided by a sister site at the South African Tuberculosis Vaccine Initiative.

There were thousands of ancillary care visits that contributed the largest number of presumptive TB cases, given the non-specific presentation of TB in infants.

#### Aeras-402 (Nochak)

The first TB vaccine trial at the site followed closely on the heels of the ICS. It was a phase IIb trial of the candidate vaccine AERAS 402 in healthy, HIV uninfected, BCG vaccinated infants [7]. The site was the first to be initiated into the trial in 2010. The study team was now proficient in recruitment of infants and requisite community links had been forged. There was widespread enthusiasm for study participation due to intense community mobilization by site staff. Locals had branded the study ‘NoChaK’ (*Nonro mar Chanjo mar Kahera*), translated ‘a new TB vaccine’ in Luo, the local language. (Supplementary Figure 2-Community Engagement).

Disappointment ensued when there was an inordinate number of screen failures due to abnormal biochemistries, particularly elevated total bilirubin in infants who were in every other sense healthy. To determine eligibility, the National Institutes of Health (NIH) reference ranges were used, which, in hindsight, were clearly derived from a distinct population. It would have been unethical to continue screening, and therefore enrolment was paused to investigate the issue. To exclude invalid laboratory results, the local laboratory that was also being used for the RTS,S malaria vaccine trial [8], was scrutinized to determine the reproducibility and repeatability of affected parameters, as well as External Quality Assurance (EQA) program results. The laboratory cleared. We found no antecedent literature describing what we were observing. Following wide consultations, we developed a new set of reference ranges. This allowed the study to proceed with enrolment. The area was hyperendemic for malaria and that was considered the prime reason for the elevations in healthy infants. Years later, two publications emerged on the subject, but for an adult population proximal to the study area, which mirrored our observations [9, 10].

There were significant problems with collection and processing of Peripheral Blood Mononuclear Cells (PBMCs). The cells needed to be separated and frozen within 4 h of collection. The study site was about 80 km away (about 1.5-h drive) from the immunology laboratory, with predictable inaccessibility in wet weather. The process of separation took at least two hours. In addition, eight ml of blood were collected in several tall vials. Like many African communities, there were strong sensitivities to blood sampling, despite our best efforts to explain that the relative blood volumes sampled would not adversely impact the babies given their age and weight. Furthermore, as infants were significantly dark skinned and healthy, if more than one attempt was needed to collect these samples, parents would get distressed and the time taken also derailed the tight separation timelines. In response, tight orchestration of the visits with PBMC collection was done. An experienced phlebotomist, efficient in cutting down the collection time was added to the team. Technicians were alerted once the sample left and were on stand-by ready to immediately begin to process the samples.

Unfortunately, the laboratory team had no prior immunology experience. They failed proficiency testing, some on more than one occasion and this skill was only gradually acquired. In addition, on one occasion the dry ice shipper containing EQA samples arrived at the destination laboratory dry, compromising cell viability. The laboratory teams were retrained, and this capacity was then available for the future trial among adults.

The stability of AERAS 402 vaccine at 2–8 degrees was short-lived. It was stored at − 80 °C in a research pharmacy in Kisumu (Supplementary Figure 3-Logistics). Twenty-four hours prior to dosing, it would be transferred from the freezer to the refrigerator and could keep for only 6 weeks. There were frequent power outages at the clinic, therefore it was not possible to safely keep vaccine on site. Therefore, an alternative thaw protocol was used. Vials would be removed from the freezer, thawed at room temperature for one to two hours, and then shipped to site under rigorous temperature monitoring. After review of potential participants, the study doctor would call up the vaccine from the central pharmacy, and vaccinations would happen upon arrival two hours later. As there were mandatory post-vaccination observation procedures, many mothers were dropped off at their homes late in the night, which created friction in families. Mothers agreed to assemble for early pick-ups on days of randomization, and thus there was only one vaccine run to Kisumu.

#### TB018 (M72)

The second vaccine trial in this site was a phase IIb trial of another candidate vaccine M72/AS01E against placebo [11]. The site’s successful experience with the MFS made it a compelling recruitment strategy for this trial. A total of 538 individuals were randomized over a period of 8 months. The laboratory was now experienced in handling immunology samples and there were few hitches.

#### VPM1002

In 2018, towards the end of the M72 study, our site was selected to participate in a phase III trial of VPM1002, a TB vaccine candidate against BCG among infants. The cumulative site experience in immunology, infant recruitment, TB case detection, and adverse event surveillance continues to be applied for this trial. Recruitment is expected to end by the second quarter of 2022, with a minimum follow-up time of 12 months.

### Lessons from site development

#### Importance of epidemiological studies

The epidemiological studies seriously enhanced our capacity to perform future trials with increasing ease and excellence. Increasingly, funding pressures have constrained the space for epidemiological studies, as they offer knowledge with no prospects of ‘return on investment’. The placebo arm is sometimes viewed as the epidemiological understudy. The trial population, however, is usually a selected sub-group that are healthier, HIV uninfected, and probably more accessible to the research team based on their different health seeking behavior. Being subjects of intense health monitoring, they likely have lower risks of TB acquisition. The placebo arm therefore provides little context for the interpretation of trial results, which are rarely linear or straight-forward. Therefore, epidemiological studies should not be considered optional.

#### Biobanking inclusivity

Health research capacity building is not without power relations [12]. Biobanking is a part of virtually every TB vaccine trial. However, once samples are shipped to central laboratories in Western Europe or North America, research teams have no say in the deployment of those assays, even while they made great efforts to collect them. It is intellectually impoverishing; research teams having the derogatory epithet ‘sample traffickers’. In hindsight, a biobanking capacity-building component should have been included in the initial studies, creating the legal and scientific framework to ensure democratization and inclusivity.

### Ebola site development

Having reflected on the development of our TB vaccine site, we turn our attention to the development of sites during the Ebola outbreak. As Ebola mortality increased during the 2014 outbreak, promising vaccine candidates stuck in preclinical phases for years [2] due to lack of funding or interest, were fast-tracked into human trials [13–15]. Clinical trial protocols were ready within weeks. Ethical and regulatory reviews were turned around in 2 weeks [16]. Preliminary efficacy results were availed in unprecedented time for rapid decision making [17]. The transition to Phase II/III studies was unmatched, at 8 months, instead of ordinarily one to 3 years [18, 19] after initiation of the phase I study [2].

To have any hope of demonstrating efficacy, the studies needed to be initiated before the unpredictable outbreak ended. However, most study areas were unmapped, and lacked electricity and basic amenities. In some places years of civil war had destroyed health infrastructure. Scores of health workers had lost their lives in the outbreak [20], and those who remained were involved in emergency response to victims. Thus, local trained research teams were virtually non-existent [21], as were any ‘clinical research sites’ [16, 21]. Despite all this, the necessary facilities were put up on the go, and teams adapted other trial aspects to the less ideal realities in the field [19].

The World Health Organization (WHO) was pivotal in overcoming all the challenges by fostering interactions with scientific, ethics, regulatory, industry and funder groups from every continent [17].

Evidently, with high stakes, absent infrastructure or research teams are not obstacles to trial implementation or success. Further, most countries with high TB burden have significantly fewer barriers to trial set-up compared to those setting up Ebola trials.

### Lessons for TB vaccine trials from Ebola

The trialing and availability of a highly efficacious vaccine for Ebola in an extraordinarily short amount of time, led us to compare the vaccine pipelines, the relative numbers of clinical trials, vaccination strategies as well as the theoretical probability of success for each disease.

#### Vaccine trial pipeline

Table 1: Ebola versus TB vaccine candidate pipeline from 2014 (WHO Vaccine Pipeline Tracker (https://www.who.int/immunization/research/vaccine_pipeline_tracker_spreadsheet/en/) accessed 12 Oct 2019). (Some trials have more than one sponsor).

**Table 1:** shows the trials initiated for Ebola versus TB vaccines in the last 5 years when Ebola vaccine trials entered the clinical phase

	Ebola vaccine trials	TB vaccine trials
**Total clinical trials**	60	30
Phase 1	34	13
Phase 2	16	10
Phase 3	8	6
Phase 4	2	1
**Sponsors**		
Industry	21	7
Non-profit	0	6
Academia	14	16
Government sponsors	17	3
a. Russia (MoH)	3	1
b. China (Jiangsu Province)	3	0
c. USA (CDC, NIAID)	11	2

We assume that a disease’s vaccine pipeline is a proxy for intensity of research and that a diverse, robust pipeline is more likely to produce a candidate that shows efficacy [21, 22]. The TB vaccine pipeline has fewer studies than does Ebola and there is also under-representation of government and industry sponsored trials. The economic cost of each epidemic makes a compelling case for both industry and government involvement. Economic losses, in West Africa and globally, were estimated at USD 18 billion as a result of the 2014–2016 outbreak [23]. The annual societal cost alone of TB is USD 19 billion [1]. The WHO has supported TB vaccine initiatives [22], but the Ebola outbreak showed us that much more is possible, especially in solving macro-level problems and mobilizing resources, probably even in sponsoring trials [16].

As of September 2019, there were only eight preclinical vaccine candidates for TB (www.tbvi.eu/what-we-do/pipeline-of-vaccines) accessed 30 September 2019. A main reason for this, other than minimal public interest in accelerating a new TB vaccine, is funding restrictions [24]. As a result, stage-gate criteria have been developed to constrict the movement of candidates from one phase to the next, with the idea of advancing only the most promising candidates, defined largely by available immunogenicity, efficacy, and safety data [21, 22]. At most of the early stage gate points, particularly pre-clinical to clinical, and Phase I to Phase IIb, the candidate has the least possible ‘value chain’ defined as the accumulation of value due to demonstrated safety or efficacy or both [23].

Furthermore, immunogenicity criteria [22] that quantify vaccine-induced immune responses may not represent protection against disease. These criteria may be flawed, leading to false negatives or positives. While clearly some criteria are needed to advance candidates, the way out seems to be through actual clinical efficacy studies.

#### Probability of success of a vaccine trial

The difficulty in vaccine design differs from pathogen to pathogen. The average vaccine requires a clinical development timeline of 10.7 years and has a market entry probability of 6% [23]. Table 2 shows selected requirements thought to impact the feasibility of accelerated vaccine development. *Mycobacterium tuberculosis* is considerably more complex, with great capacity to evade host immunity [25]. Also, animal models that closely mimic human disease are better for Ebola virus than TB. Hence a new TB vaccine could be termed ‘unprecedented’ and the odds of success are pegged considerably lower than for most other diseases [24]. These estimates are valid but are based on past experience with limited government and industry involvement and a minimal global effort.

**Table 2:** Feasibility of accelerated vaccine development (Institute of Medicine (US) Committee on Issues and Priorities for New Vaccine Development [26]

Requirement	Ebola Virus	Tuberculosis
knowledge of clinical signs and symptoms of the disease to allow differentiation from similar syndromes	+	++
Knowledge of pathogen characteristics: strains, serotypes, infectivity, virulence, antigenicity, immunogens	++ [27]	++ [28, 29]
Strains & Serotypes		
Infectivity and Virulence		
Antigenicity		
Immunogens		
Ability to cultivate pathogen	+ [30]	++
Identified non-human models of infection, closely mimicking human disease	+++ [31]	++ [32]
Knowledge of human immune response to the pathogen (duration, type of response)	+ [33]	+ [34]
Definition of the target population	+ [35]	++ [36]

#### Vaccination strategies and phase II/III trial sites

Ring vaccination in the Guinea Ebola vaccine trial provided preliminary efficacy data in a highly dynamic outbreak [2]. The most at risk persons were singled out for vaccination, as opposed to vaccinating an entire community.

There are irrefutable data on asymmetries of TB risk (‘TB hotspots’) [37, 38] based on residence (urban vs. rural) [39, 40], certain geographic locations including prison [41] and mines [42], social class interactions [43], and age [44, 45]. Such epidemiological data have not been applied in selecting trial populations for phase IIb-IV trials, yet they would critically minimize sample sizes, guide site selection and provide rapid answers as to a vaccine’s efficacy. This was one of the pathways explored in the Ebola outbreak.

## Conclusions

We have described our incremental 12-year process of site development, in contrast to an acute, chaotic and very productive process in the Ebola virus outbreak. Since the ACS in 2007, about a dozen other phase II and III trials have been conducted at our site. Subsequent studies have found better infrastructure, more highly experienced teams, and the complexity of protocols implemented at the site has also increased. Nevertheless, the end goal is a new efficacious TB vaccine brought to bear on the epidemic.

TB unlike Ebola has a lower case fatality, slower epidemic, and lower potential for bioterrorism. But its total mortality is hundreds to thousands of times higher. It is our view that developing a new TB vaccine will also require centralized coordination of efforts.

The TB vaccine pipeline is plagued not simply by funding limitations, but by an inadequate number of ‘hands on deck’. The Ebola crisis calls for a sense of urgency towards the TB epidemic, and for a narrowed focus. For example, the lack of knowledge of correlates of protection has not prevented the advancement of a new malaria, polio [46], or Ebola vaccine [47, 48], neither the eradication of small pox [49]. Funds can be prioritized for pre-clinical development and field testing of candidates in order to stop the scourge, else the goal of TB elimination will remain elusive [1]. Given an Ebola vaccine was evaluated and licensed under extreme conditions, it is a rallying call for the TB vaccine community to champion the cause.

## Supplementary information

**Additional file 1: Figure S2.** Community engagement.

**Additional file 2: Figure S3.** Logistics.

**Additional file 3: Figure S4.** Siaya Clinical Research Annex.

**Additional file 4: Figure S5.** Mobile Field Site.

## Data Availability

Not Applicable.

## Abbreviations

ACS: Adolescent Cohort Study
BCG: Bacille Calmette Guerin
EDCTP: European and Developing Countries Clinical Trials’ Partnership (EDCTP)
EQA: External Quality Assurance
HDSS: Health and Demographic Surveillance System
HIV: Human Immunodeficiency Virus
ICS: Infant Cohort Study
GCP: Good Clinical Practice
MFS: Mobile Field Site
National Institutes of Health: National Institutes of Health
Nochak: NOnro mar CHAnjo mar Kahera
TB: Tuberculosis
TST: Tuberculin Skin Test
TBA: Traditional Birth Attendant
PBMCs: Peripheral Blood Mononuclear Cells
USD: United States Dollar
WHO: World Health Organization
