# Supplementary figures and images for: Development of a TB vaccine trial site in Africa and lessons from the Ebola experience
Source: BMC Public Health. 2020 Jun 26;20:999. doi: 10.1186/s12889-020-09051-3 (PMC7316575; doi:10.1186/s12889-020-09051-3)

Supplementary Figure 2: Community engagement


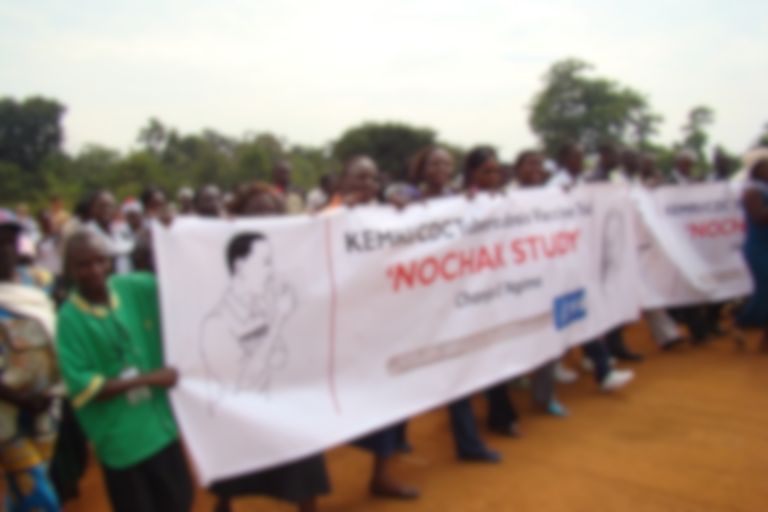

Supplement: Supplementary file 1 — Additional file 1: Figure S2. Community engagement. [file 12889_2020_9051_MOESM1_ESM.docx]

Figure 4: Siaya Clinical Research Annex


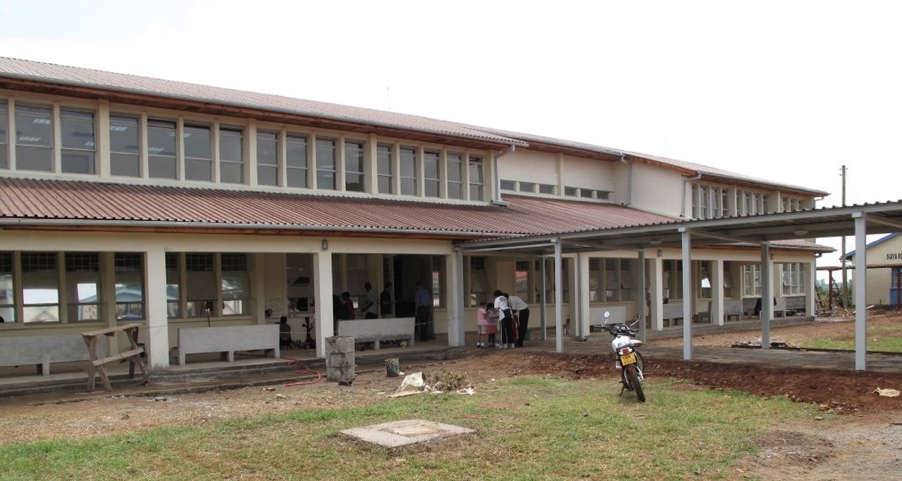

Supplement: Supplementary file 3 — Additional file 3: Figure S4. Siaya Clinical Research Annex. [file 12889_2020_9051_MOESM3_ESM.docx]

Figure 5: Mobile Field Site


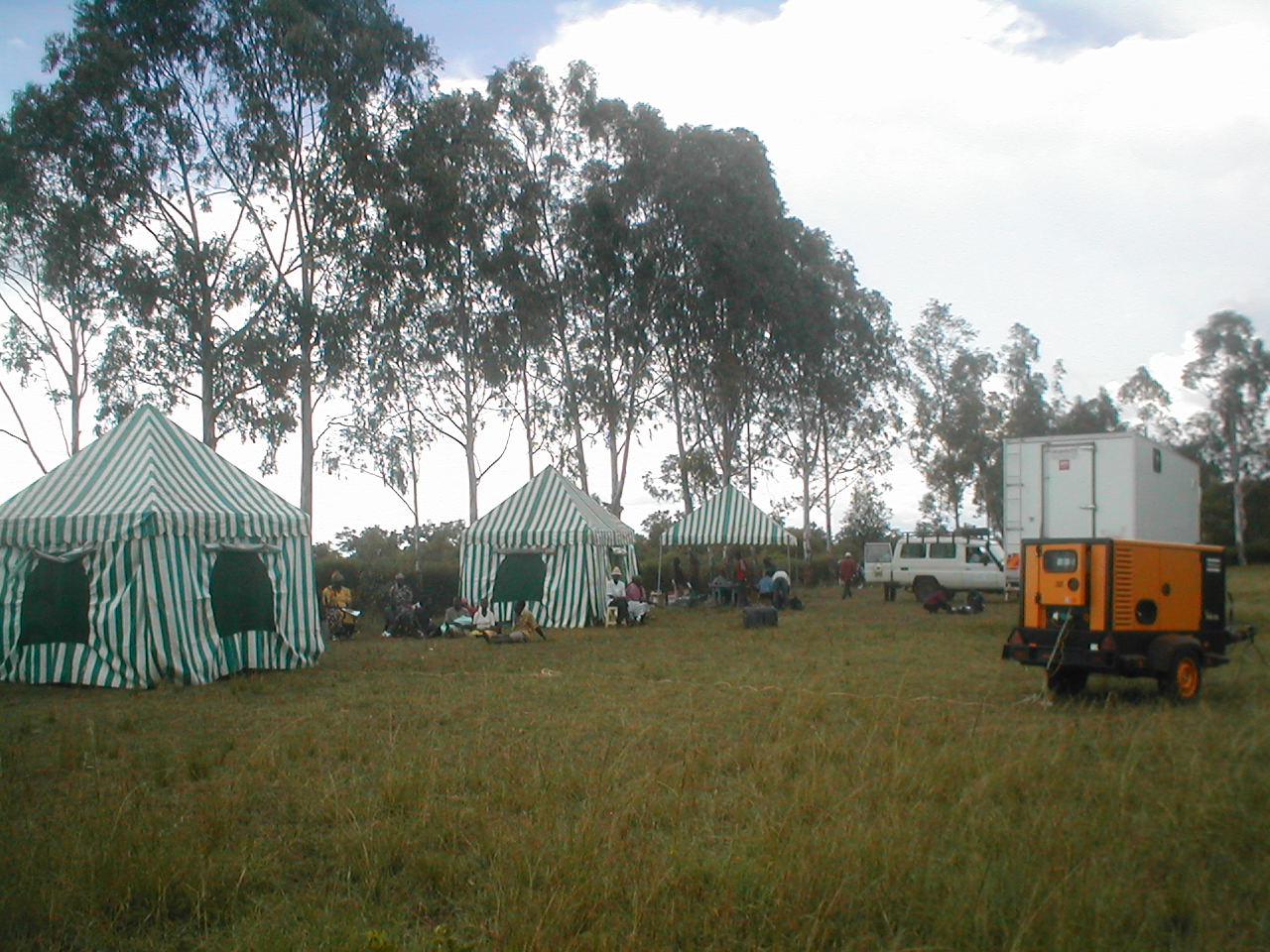

Supplement: Supplementary file 4 — Additional file 4: Figure S5. Mobile Field Site. [file 12889_2020_9051_MOESM4_ESM.docx]
