# Supplementary material for: Development of a TB vaccine trial site in Africa and lessons from the Ebola experience
Source: BMC Public Health. 2020 Jun 26;20:999. doi: 10.1186/s12889-020-09051-3 (PMC7316575; doi:10.1186/s12889-020-09051-3)
Supplement: Supplementary file 2 — Additional file 2: Figure S3. Logistics. [file 12889_2020_9051_MOESM2_ESM.docx]

Supplementary Figure 3: LogisticsDBS: Dry Blood Spot

PBMC: Peripheral Blood Mononuclear Cells
